# Social recovery in substance use disorder: A metasynthesis of qualitative studies

**DOI:** 10.1111/dar.13434

**Published:** 2022-02-01

**Authors:** Mariann Iren Vigdal, Christian Moltu, Jone Bjornestad, Lillian Bruland Selseng

**Affiliations:** ^1^ Department of Welfare and Participation Western Norway University of Applied Sciences Sogndal Norway; ^2^ District General Hospital of Førde Førde Norway; ^3^ Department of Health and Caring Sciences Western Norway University of Applied Science Førde Norway; ^4^ Department of Social Studies University of Stavanger Stavanger Norway

**Keywords:** substance use disorder, social recovery, social community, systematic review, metasynthesis

## Abstract

**Issues:**

In substance use disorder, connection to social communities plays a significant role in the recovery process. The aim here has been to identify and synthesise the qualitative research examining the process of social recovery from a first‐person perspective and how social communities assist in this process.

**Approach:**

Metasynthesis using the following databases: CINAHL, Embase, MEDLINE, PsycINFO, Scopus, SocIndex and Web of Science. The search returned 6913 original articles, of which 18 met the following criteria: examining the experience of social recovery from a first‐person perspective and how social communities support this process, age of 18+, recovery of at least 12 months, in an English‐language peer‐reviewed journal. Review protocol registration: PROSPERO (CRD42020190159).

**Key Findings:**

The persons in recovery emphasised communities that they perceived as being safe and non‐stigmatising. These are qualities that contributed to positive self‐change, and these communities were perceived as suitable arenas in which to confront responsibility and trust. Additionally, participants found that their relationship skills were improving due to the new social bonds forged in these communities. A sense of citizenship was gained along with a regaining of social dignity through voluntary work and giving back to society.

**Implications:**

The pivotal role of the social community identified in this review underscores the importance of recognising and supporting persons in recovery's needs when connecting with such communities

**Conclusion:**

We propose a four‐stage model to guide research into social recovery from a first‐person perspective and how social communities support this process.

## Introduction

Connection with social communities plays an essential role in the recovery process following a substance use disorder (SUD) [1, 2, 3, 4, 5, 6]. SUD recovery is described as a long‐term recovery (LTR) process that involves personal and social changes that are unique to the individual human being [2, 3, 7, 8, 9]. In order to approach SUD recovery as a process of social change, it is necessary to recognise the value of interaction between persons and to view everyday life as an essential arena for recovery [2, 3, 10, 11]. Recovery is described as ‘a process of restoring a meaningful sense of belonging to one's community and positive sense of identity apart from one's condition while rebuilding a life despite or within the limitations imposed by that condition’ [5, p. 25]. In this article, we operationalise social community as a human system given form through interactions, conversations or activities that build relatedness [12, 13, 14].

One systematic review has emphasised the building personal relationships and a sense of community belonging as facilitators in the recovery process [15]. Stigma is a prominent obstacle to community participation in SUD recovery [2, 16, 17]. Stigma refers to an attribute that is deeply discrediting for the individual [18]. Stigma and exclusion can be challenged by participating in activities that are experienced as meaningful [19]. However, this can be difficult, as stigma, self‐stigma and discrimination are barriers to developing interpersonal relationships and thus pose a significant challenge to those in SUD recovery [16, 17, 20, 21].

From a relational recovery perspective, interpersonal relationships and social contexts are seen as prerequisites for recovery [6]. In this article, social recovery is operationalised as ‘people's ability to lead full and contributing lives as active citizens’ [22, p. 360]. There is an ongoing discussion in the field about the need for abstinence from substance use in the LTR process [23]. A growing body of evidence shows that moderate use of a substance is possible among many of those who have achieved a sustained reduction in the frequency of substance use [23]. Such recovery literature emphasises the relevance of focusing more on the importance of ‘people's ability to lead full and contributing lives as active citizens’ [22, 23]. In personal recovery, connectedness is highlighted as a pivotal process that is interconnected with quality of life, hope and optimism regarding the future, identity, meaning in life and empowerment [6, 23, 24, 25].

A review of studies on the relationship between Alcoholics Anonymous (AA) and social networks illustrates the importance of social communities in LTR [1]. AA had its greatest impact on the development of close friendships. Furthermore, through friends in AA, skills were being taught that were necessary to maintain abstinence [1]. A review of studies on first‐person perspectives on facilitators in and barriers to dual recovery supports these findings [2]. A meaningful everyday life, including re‐establishment of one's social life, supportive relationships and a sense of being a productive citizen were found to be a key facilitator of overcoming loneliness, boredom and sustained recovery [2].

One systematic review showed that, although social issues are critical factors in LTR, only seven (1.4%) out of 504 studies have reported outcomes on social factors such as friendship, support and social relationships [7]. Furthermore, there is a lack of high‐quality longitudinal studies with a follow up of at least 2 years that investigate social recovery within LTR [7]. Paradoxically, there are shortcomings in what is known, although social recovery within LTR is a target of interventions [7, 15, 26, 27]. One systematic review concludes that the role of broader society is discussed in the literature to only a very limited degree [15]. In order to accommodate this knowledge gap, we aim to identify and synthesise the qualitative research examining the process of social recovery from a first‐person perspective and how social communities assist in this process.

## Methods

The method selected for this study is qualitative metasynthesis [28]. A qualitative metasynthesis is ‘the process and outcome of organizing and interpreting research findings about a particular matter, leading to new, conceptual understanding beyond the average or sum of parts’ [29, p. 7]. Our metasynthesis was performed in three steps to secure comprehensive and transparent reporting on methods and results [30]. The PRISMA guidelines [31] were applied to the search strategy and data extraction. The Critical Appraisal Skills Programme (CASP) checklist was used for quality appraisal and final study inclusion [32]. Data analysis followed a structured framework for qualitative metasynthesis [28]. The study protocol was registered with the PROSPERO International prospective register of systematic reviews in July 2020 (registration no. CRD42020190159).

### Sample

**Figure 1:**
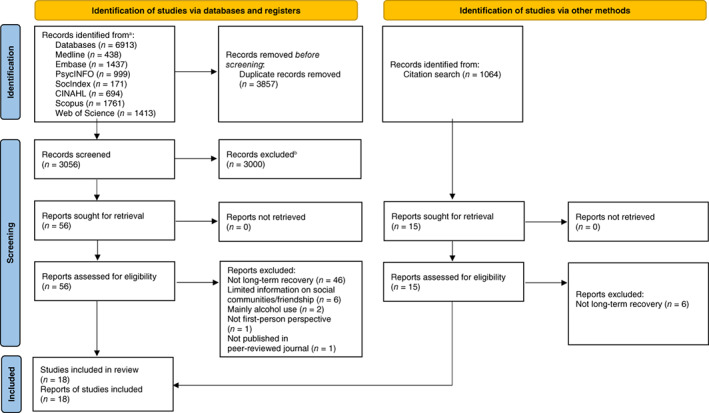
PRISMA 2020 flow diagram for new systematic reviews that include searches of databases, registers, and other sources. ^a^Consider, if feasible to do so, reporting the number of records identified from each database or register searched (rather than the total number across all databases/registers). ^b^If automation tools were used, indicate how many records were excluded by a human and how many were excluded by automation tools. (Adapted from Page *et al*. [33], with permission. For more information, visit: http://www.prisma‐statement.org/.)

Systematic literature searches were performed on 16 April 2021 using the following databases—CINAHL, Embase, MEDLINE, PsycINFO, Scopus and SocIndex—and on 19 April 2021 using Web of Science. This literature search included the following four elements: SUD, recovery, quality of life and social communities. Each element was searched for by subject heading and other search terms (text words) and then combined with the Boolean operator ‘AND’, which requires all elements to be present. The search was developed in order to search EMBASE with an Ovid interface (see Figure 1). The subject headings and thesaurus terms were adapted for each of the remaining databases that have a thesaurus. The text words searched were the same for all databases. The inclusion criteria were: (i) age of 18+; (ii) being in recovery for at least 12 months from a SUD, operationalised as abstinence from drugs; (iii) studies examining the process of social recovery from a first‐person perspective and how social communities assist in this process; and (iv) publication in the English language in peer‐reviewed journals. Exclusion criteria for the studies were studies that identified themes relating to tobacco or alcohol only.

### Procedures

All references from the search were imported into EndNote and duplicates were removed. MIV performed the screening of all the potential studies. Three independent reviewers (CM, JB and LBS) each performed a separate screening of one‐third respectively of the titles and abstracts. Based on this screening, the reviewers suggested a list of articles for full‐text review. MIV performed the full‐text reading of all potential studies. The independent reviewers (CM, JB and LBS) each performed a separate full‐text reading of one‐third of these respectively. Five consensus meetings were arranged where the relevance of the articles was assessed after the full‐text reading. In the end, all of the reviewers made a joint assessment of all of the full‐text studies together. We used the CASP checklist for quality appraisal and final study inclusion [32].

### Analyses

A thematic analytic procedure building on the metasynthesis theory of Noblit and Hare [34] and revised by Malterud [28] was used to implement the meta‐analysis. A first‐order analysis may be considered as the result section in the included primary studies [35, 36]. Our analysis is a second‐order analysis, which we began by closely reading the results sections of all of the studies included and then synthesising the results of the first‐order analysis [35, 36]. We identified preliminary themes in the primary studies which became our point of departure for a systematic second‐order analysis: (i) the function of social communities; (ii) the characteristics of the social communities; and (iii) the conditions for utilising these social communities. We chose Best and Gow [37] as our index study, as their study was characterised by methodological quality and rich data. First, the empirical material was organised into a matrix of relevant topics and metaphors from each primary study were listed in vertical columns [28]. Second, we organised the selected texts together with related themes and metaphors from all of the studies in the horizontal rows of the matrix, placing an emphasis on similarities and differences in conceptual use. We looked for both thematic convergence and divergence in the results section of the studies included. Third, we reviewed each of the horizontal rows to develop an overall abstraction encompassing all of the themes and metaphors in the form of a new phrase that provides an original and independent understanding of the findings. Convergences were, for example, the functions and characteristics of the social communities. Fourth, we elaborated upon the meaning of the expressions derived from the synthesis to produce an understanding (e.g. the importance of connecting with social communities).

## Results

### Search results

The electronic search returned 6913 articles. After the duplicates were removed, 3056 articles remained. Three thousand articles were excluded after titles and abstracts were screened. We identified 15 studies through by way of a citation search. A full‐text evaluation was then conducted for 71 articles, of which 18 remained for the final analysis. See Figure 1 for search details.

### Quality appraisal and study characteristics

All the articles received satisfactory scores based on the CASP assessment [32], 10 of which received full scores (see Table 1). Reflexive and ethical issues achieved the lowest scores. Across the studies, a total of 523 participants were included (range: 5–205) (see Table 2). Participant ages ranged from 18 to 78 years, and the studies were mainly within Caucasian cultures. Approximately 45% of the participants were female. It was not possible to obtain a precise number due to the lack of gender reporting in one of the studies. All the included papers comprised experience of SUD recovery and the average time in recovery was 7.3 years. Although all of the included studies indicated that the participants had been abstinent for 12 months or longer, different interpretations of the meaning of ‘abstinent’ may have led to different inclusion criteria (see Table 2). The inclusion criteria did not relate to the type of social community. However, it turned out that all of the studies included described experience from either Narcotics Anonymous (NA) or AA communities (i.e. 14 of the studies) [37, 38, 39, 42, 43, 44, 45, 46, 47, 49, 50, 51, 52, 53] or from religion‐based communities (7 of the studies) [40, 41, 43, 45, 48, 50, 53], or else described the maintenance of relationships from before and during the periods of substance use (4 of the studies) [37, 40, 41, 46].

**Table 1:** Quality appraisal of the 18 articles (quality criteria: see Critical Appraisal Skills Programme criterion)

		1.	2.	3.	4.	5.	6.	7.	8.	9.	10.
	Study	Clear statement of the aims of the research?	A qualitative methodology was appropriate?	Research design appropriate for the aims of the research?	Recruitment strategy appropriate for the aims of the research?	Data collected in a way that addressed the research issue?	Relationship between researcher and participants was adequately considered?	Ethical issues taken into consideration?	Data analysis sufficiently rigorous?	Clear statement of findings?	Is the research valuable?
1.	Abram and Jane [38]	Yes	Yes	Yes	Yes	Yes	Cannot tell	Yes	Yes	Yes	Yes
2.	Best *et al*. [37]	Yes	Yes	Yes	Yes	Yes	No	Yes	Cannot tell	Yes	Yes
3.	Bjornestad *et al*. [39]	Yes	Yes	Yes	Yes	Yes	Yes	Yes	Yes	Yes	Yes
4.	Blount *et al*. [40]	Yes	Yes	Yes	Yes	Yes	Yes	Yes	Yes	Yes	Yes
5.	Cloud and Granfield [41]	Yes	Yes	Yes	Yes	Yes	No	Cannot tell	Yes	Yes	Yes
6.	Elswick *et al*. [42]	Yes	Yes	Yes	Yes	Yes	No	Yes	Yes	Yes	Yes
7.	Flaherty *et al*. [43]	Yes	Yes	Yes	Yes	Yes	No	Yes	Yes	Yes	Yes
8.	DeLucia *et al*. [44]	Yes	Yes	Yes	Yes	Yes	Yes	Yes	Yes	Yes	Yes
9.	Grant [45]	No	Yes	Yes	Yes	Yes	No	Cannot tell	Cannot tell	Yes	Yes
10.	Gueta *et al*. [46]	Yes	Yes	Yes	Yes	Yes	No	Yes	Yes	Yes	Yes
11.	Gueta and Addad [47]	Yes	Yes	Yes	Yes	Yes	No	Yes	Yes	Yes	Yes
12.	Kang *et al*. [48]	Yes	Yes	Yes	Yes	Yes	Yes	Yes	Yes	Yes	Yes
13.	Moghanibashi‐Mansourieh *et al*. [49]	Yes	Yes	Yes	Yes	Yes	Yes	Yes	Yes	Yes	Yes
14.	Nehlin *et al*. [50]	Yes	Yes	Yes	Yes	Yes	Yes	Yes	Yes	Yes	Yes
15.	Pettersen *et al*. [51]	Yes	Yes	Yes	Yes	Yes	Yes	Yes	Yes	Yes	Yes
16.	Rettie *et al*. [52]	Yes	Yes	Yes	Yes	Yes	Yes	Yes	Yes	Yes	Yes
17.	Stokes *et al*. [53]	Yes	Yes	Yes	Yes	Yes	Yes	Yes	Yes	Yes	Yes
18.	Veseth *et al*. [54]	Yes	Yes	Yes	Yes	Yes	Yes	Yes	Yes	Yes	Yes

**Table 2:** List of included studies

Study	Country	Study information	Average time in abstinence	Data collection	Participants	Mean age, years
Abram and Jane [38]	USA	All participants were Caucasian and had used alcohol as well as other substances such as cocaine and heroin. Self‐defined recovery. Participants were abstinent from 18 months to 7 years	3.6 years	Semi‐structured interviews	8 participants 3 males	Range 28–53
Best *et al*. [37]	Scotland	The majority (*n* = 199, 97.1%) were white British. Half of the participants were in recovery from alcohol and the others were in recovery from heroin dependence. Participants had not used that primary substance in the previous 12 months. For alcohol participants, this was an average of 8 years from the time they started their recovery. For heroin participants, this was an average of 4 years from the time they began their recovery process	6 years	Semi‐structured interviews and self‐completed questionnaires	205 participants 137 males	43.2
Bjornestad *et al*. [39]	Norway	All participants were native Norwegian. 20 participants had 5 years of abstinence and 10 participants had 4 years of abstinence	4.7 years	Semi‐structured interviews	30 participants 17 males	25.9
Blount *et al*. [40]	USA	All eight participants were African American women who had been abstinent for 5 to 30 years	At least 5 years	Semi‐structured interviews	8 female participants	61
Cloud and Granfield [41]	USA	The sample included 43 Caucasians, one African American and two Hispanics. Natural recovery. Participants had resolved their dependence for a period of at least one continuous year. The mean length of cessation of alcohol (*n* = 25) was 6.8 years and for substance use (*n* = 21) this was 5.9 years	6.4 years	Semi‐structured in‐depth interviews	46 participants 28 males	38.4
Elswick *et al*. [42]	USA	The 8 participants in this study were White and predominantly middle to upper‐middle class. The participants had been in recovery for at least 12 months and did not have medication‐assisted treatment	At least 1 year	Semi‐structured interviews	8 participants 4 males	Range 18–25
Flaherty *et al*. [43]	USA	Five participants were White and one participant was African American; all had at least 5 years of self‐identified recovery	15.8 years	Semi‐structured interviews	6 participants 5 males	No information
DeLucia *et al*. [44]	USA	Most participants identified as Caucasian, 19% identified as a member of an ethnic minority group	19.5 years	A survey and focus groups	21 participants 12 males	53.4
Grant [45]	USA	The participants were rural Appalachian women. Participants' drug(s) of choice included alcohol (13) and alcohol and marijuana (7). Minimum 18 months or more of abstinence	8 years	Semi‐structured interviews	25 female participants	40
Gueta *et al*. [46]	Israel	A mixed‐methods design was employed among 229 respondents with a qualitative subsample of 41 participants. All participants had been abstinent for a minimum of 12 months. Their country of birth was Israel, Ethiopia or the former USSR	1 year	In‐depth interviews	41 participants No information about the sex	Range 22–49
Gueta and Addad [47]	Israel	All research participants were Jewish Israeli women; 8 were born in Israel and 6 were born in the former Soviet Union. Participants had been abstinent from 2 to 7 years	At least 2 years	Semi‐structured in‐depth interviews	9 female participants	Range 22–46
Kang *et al*. [48]	Korea	Participants' drug(s) of choice included methamphetamine (3), marijuana (1), and cocaine/ecstasy (1). Participants had been abstinent from 4 to 8 years	6 years	Semi‐structured interviews	5 participants 3 males	41
Moghanibashi‐Mansourieh *et al*. [49]	Iran	The participants were from Iran and had been abstinent for at least 2 years	8.6 years	Semi‐structured interviews	27 male participants	47.6
Nehlin *et al*. [50]	Sweden	The majority of the Swedish participants had been drug‐free for 20 years or more. One had been drug‐free for 1.5 years	16.6 years	Semi‐structured interviews	11 participants 6 males	47.8
Pettersen *et al*. [51]	Norway	Six participants had mainly used heroin, 5 had mainly used alcohol, 5 had a history of mixed use of several substances, 1 had used only amphetamines, and 1 had used only cannabis. Eight participants were completely abstinent at the time of the interview, while 10 reported unproblematic alcohol use. Participants had a minimum of 5 years of abstinence from problematic substance use	12 years	Semi‐structured qualitative interviews	18 participants 10 males	54
Rettie *et al*. [52]	North Wales	The participants from North Wales had been in recovery between 7 months and 9 years	3.2 years	Semi‐structured interviews	10 participants 7 males	No information
Stokes *et al*. [53]	South Africa	Participants were resident in the Gauteng area of Pretoria and Johannesburg, South Africa and had been abstinent for a minimum of 3 years. Twelve had 3–9 years of abstinence. Two had been abstinent for more than 10 years. One had been abstinent for 41 years	6.5 years	In‐depth interviews	15 participants 9 males	Range 25–78
Veseth *et al*. [54]	Norway	All participants were native Norwegians. 20 participants had been abstinent for 5 years and 10 participants had been abstinent for 4 years	4.7 years	Semi‐structured interviews	30 participants 17 males	25.9
			7.3 years (mean)		Sum: 523 participants	

### Meta‐themes

The analysis resulted in four meta‐themes comprising the first‐person experiential domains of: (i) social communities perceived as safe and non‐stigmatising and contributing to self‐change; (ii) arenas in which to confront responsibility and trust; (iii) forging new social bonds in social communities; and (iv) regaining social dignity by giving back to society. See Table 3 for a summary of how the studies contribute to the different meta‐themes.

**Table 3:** List of papers contributing to the meta‐themes

Contributing papers	Social communities perceived as safe and non‐stigmatising and contributing to self‐change	Arenas in which to confront responsibility and trust	Forging new social bonds in social community	Regaining social dignity by giving back to society
Abram and Jane [38]	X	X	X	X
Best *et al*. [37]	X		X	X
Bjornestad *et al*. [37]	X	X	X	
Blount *et al*. [40]	X	X	X	X
Cloud and Granfield [41]	X	X	X	X
Elswick *et al*. [42]	X	X	X	
Flaherty *et al*. [43]	X	X	X	X
DeLucia *et al*. [44]	X	X	X	X
Grant [45]	X	X	X	X
Gueta *et al*. [46]	X	X	X	
Gueta and Addad [47]	X	X	X	
Kang *et al*. [48]		X	X	X
Moghanibashi‐Mansourieh *et al*. [49]	X	X	X	
Nehlin *et al*. [50]	X	X	X	X
Pettersen *et al*. [51]	X	X	X	
Rettie *et al*. [52]	X		X	X
Stokes *et al*. [53]	X	X	X	X
Veseth *et al*. [54]		X	X	X

#### Social communities perceived as safe and non‐stigmatising and contributing to self‐change

All studies emphasised the essential role of social communities in the development of self‐change. Persons in LTR emphasised the importance of social communities, regardless of whether they participated in groups such as NA [37, 38, 39, 42, 43, 44, 45, 46, 47, 49, 50, 51, 52, 53] or religious communities [40, 41, 43, 45, 48, 50, 53] or whether they were self‐changers [37, 40, 41, 46]. Self‐changers (i.e. those who changed without aid from substance use treatment or a mutual aid group) described being able to maintain relationships with family and friends during the period of substance use [41]. Participants perceived that they were safe and non‐stigmatised in the communities referred to above [37, 38, 39, 40, 41, 42, 43, 44, 45, 46, 47, 49, 50, 51, 52, 53]. The atmosphere in NA communities was characterised as warm, welcoming, inclusive and open towards persons in recovery: ‘You don't have to hide behind a mask, you can tell them how you feel’ [52, p. 6]; ‘It's the best thing I've been in because it's people like me’ [37, p. 12]. These social communities of like‐minded persons were described as safe places where one could feel accepted and welcome despite their substance use issues [44, 52]. A participant described how she experienced the group atmosphere: ‘I feel safe because the people in there don't mean me no harm, they don't want nothing off me, they don't want me to use, they want the best for me, they love me’ [52, p. 7]. Participants perceived a high social tolerance for diversity and symptom severity, which was absent in other social settings [44]. Such open and inclusive attitudes provided an experience of connection and gradually of belonging to the community [37, 39, 40, 43, 44, 45, 47]. Participants referred to recovery as internal self‐work and external change with which they needed to engage [38]. Both internal self‐work and external change involved change that influenced their selves [38].

Human closeness within the community may be a more important community function than the actual content discussed [52]. The freedom to discuss any topic and come and go as one pleases was a characteristic of the NA communities [52]. Regardless, six studies show that persons in LTR at some point experienced a different view of communities such as NA [37, 38, 39, 43, 45, 52]. After the early stages of recovery, participants were more likely to talk about moving on and leave the community of former substance users [37, 38, 39, 43, 45, 52]. Some indicated that they did not identify the social community as a vital influence within their recovery anymore [52]. A Scottish participant explained how he had changed his view about the community: ‘I've got long past [the] early stages of recovery, I don't see myself as an addict anymore, I don't see addiction as an illness anymore’ [37, p. 9].

Taken together, a common denominator of the various environments is the importance of being safe and not being stigmatised, regardless of whether it was a NA or religious community or family and friends with whom one maintained contact throughout the period of substance use.

#### Arenas in which one can confront responsibility and trust

Most of the participants referred to the social communities as an arena in which they could confront responsibility and trust. Sixteen of the studies thematised how demanding it was to seek new social communities [38, 39, 40, 41, 42, 43, 44, 45, 46, 47, 48, 49, 50, 51, 53, 54]. Showing vulnerability was viewed by participants as a weakness that involved the self's exposure and shame [38]. One study emphasised that hope for a different and better life helped participants to move forwards in the process [38]. When participants moved backwards, this was due to fear—for example, fear of face‐to‐face exposure [38]. ‘I was consumed by fear, and a self‐loathing, I had to allow myself to experience emotions—to be okay to feel this way—not to run away or pull back, being okay with being uncomfortable’ [38, p. 7]. Participants who had a relapse in the past acknowledged their need to show weakness and face exposure and vulnerability in order to achieve sustained recovery [38].

The process of establishing oneself in a new social environment required that individuals be willing to show up where others were gathered [41, 44]. This meant establishing new routines and becoming familiar with new roles and relationships and everyday life [41, 53]. One participant from Scotland described it this way:

*‘But it's not only about wanting it, it is about* [being] *willing to make the effort to get it, and the thing is, you've got to be responsible in here … And with that responsibility, you learn the value of what you have in your life’* [44, p. 11].

The participants experienced routines that consisted of planning activities and striving for new relationships [45]. As a Norwegian participant put it: ‘Beginning to take care of my relationships with other people again was hard, but still, very important’ [51, p. 4]. Participants found it difficult and almost unthinkable to trust others [39, 43]. A Norwegian participant described this as a process of change where they had to construct a new mindset with which they could look at other persons as individuals they could trust [39]:

*‘That's what costs me more than any of it almost, out of all I have been through, it is in a way working with letting people in and caring about people, and letting them care about me and … so I haven't sort of been ready for that either, until now’* [39, p. 5].

Acknowledging a need for social dependence was highly anxiety‐provoking [39]. Any form of strong emotion, whether joy or sorrow, often triggered a desire to use drugs instead of a need for relational closeness [39]. Prolonged recovery required a new self‐attitude and a view of oneself in which self‐acceptance grew and self‐stigma was reduced [39, 45].

This improved self‐esteem provided coping strategies for dealing with stressors in life [47, 53].

#### Forging new social bonds in social communities

Time and space were dedicated to practical guidance on how to master social life in LTR. Participants experienced the NA communities as social meeting places where persons in recovery could interact, thus forging new social bonds, and experience being seen, accepted and recognised by others. All of the studies included described participants in LTR as switching from social communities dominated by active users to ones where individuals are abstinent [37, 38, 39, 40, 41, 42, 43, 44, 45, 46, 47, 48, 49, 50, 51, 52, 53, 54]. Some self‐changers indicated that they continued to have friends who used drugs [43, 46].

Persons in LTR chose to be social and did activities with others who were non‐users [37, 41, 44, 52, 53]. However, while changing one's social community, one may feel sad, lonely and isolated [54]. One Norwegian participant said the following in respect of how he felt after he left a network dominated by active users:

*‘You've cut yourself off from your whole group, you've dropped your friends, there's nothing left. So you sit there and wait for the alarm to go off so can go to work again the next day. Nobody can handle that in the long run! What's the point really? And most people who've been getting high for 10, 15, 20 years, they've pushed away all their family and friends and such for the high, everyone who was responsible and upstanding. They're gone from their life, because they've disappeared in the high*’ [54, p. 9].

Participants referred to social support, especially peer support, as a key factor in sustaining recovery [37]. They perceived social communities as an essential function as they are an arena in which to be socialised into new communities [37, 38, 39, 44, 45, 48, 52, 53, 54].

The communities provided structure and filled every day with meaningful content [52, 53]. Persons in LTR described the communities as an important tool for cultivating hope, connection, belonging and fun in recovery [44]:

*‘I think that's such a key thing … the acceptance and the understanding, not just of the sponsor, but of all of us towards each other. I mean I remember when I first started coming to meetings. It's like the first place I ever actually felt at home’* [44, p. 12].

Participants described the importance of keeping busy and involved in meaningful activities in order to sustain recovery [37, 41, 43, 44, 45, 46, 50, 52, 53]. A South African female expressed how important it was for her to keep herself busy:

*‘So I managed to white knuckle it* [figure of speech used in the recovery community to indicate holding on for dear life and resisting using substances] *for that first year, slept a lot, did a lot of meetings, went for coffees, but I didn't go to any parties, which is where I used to use*’ [53, p. 7].

The communities allowed participation in fun and prosocial activities in low‐risk contexts [44]. A participant from one study in the USA put it like this:

*‘What I really liked was that there were opportunities to have fun in my area through camping, dances, etc. We had something to do other than getting high on a Friday or Saturday night or a holiday’* [44, p. 13].

In these communities, participants were supported in living life without using drugs [37, 44, 45, 52]. There are several dimensions to this support; it can take the form of practical help, financial support [40], advice and encouragement [37], and/or guidance through complicated feelings and situations [44]. In particular, social contact with other persons is mentioned as necessary [37, 44]. Others within social communities acted as role models for those in an earlier stage of their recovery process [37, 39, 44, 45], for whom belief and hope that recovery was possible were created by watching and listening to these others' life stories [37, 44, 51]. One participant from the USA discussed how the community inspired him in his recovery process:

*‘People within the group are verbalising their experience, strength, and hope. They are talking about what they are going through, not just their problems but their solutions. And that gives you the hope, and that hopefully will trigger the willingness for you to model after them’* [44, p. 11].

#### To regain social dignity by giving back to society

There was a clear pattern of participants feeling the need to regain their social dignity during LTR. Persons in LTR regain social dignity by giving back to peers and doing voluntary work in society. Twelve of the studies reported positive experiences of giving back by way of voluntary work [37, 38, 40, 41, 43, 44, 45, 48, 50, 52, 53, 54]. A participant from Korea who had stopped using drugs for 7 years discussed how he had regained his dignity through voluntary work:

*‘People call me “Head”, but I am actually an errand boy of this village. I do my best because helping the elderly in my village is helping myself, as well, because I respect them. Moreover, although being the head of the village is nothing special, as the head, I am dignified, so I cannot smoke*’ [48, p. 8].

Through the interaction with others in social communities, there was a gradual change in the person's experience of self‐acceptance and self‐worth. Participants discussed how contributing to voluntary work affected their self‐confidence in recovery, made them less self‐absorbed, and helped them change their perception of themselves [37]. ‘For the first time in my life I'm actually starting to like myself’ [37, p. 12]. Voluntary work was perceived as improving a person's self‐esteem in recovery [37]. ‘I started doing voluntary work in a wee project that I came through and that's been really good … it's good for my self‐esteem, which was really low before’ [37, p. 12]. Participants perceived social communities as offering a valuable opportunity for social training and volunteering and as a form of support and resource for others in similar situations [37, 38, 39, 44, 45, 52, 53]. One study found that relationship skills were developed within the context of recovery‐based relationships and that participants found that they were able to use these skills in respect of family and friends outside of NA [44]. Some studies found that volunteering can provide a sense of citizenship [39, 44] and a broader sense of being fully included as a member of society [39]. At the same time, voluntary work was seen as a way of escaping social isolation [48]. Peer communities act as a catalyst for one to participate in other groups and expand one's social network [52]. ‘I think it gave me more confidence as well […] because I've ended up going off with other groups and there's been a knock on effect’ [52, p. 6]. Participants talked about doing voluntary work with great pride [44, 50, 52] and with gratitude for being accepted and for society needing them [39]. The act of giving back served as a reminder of where the participants had come from [38, 52]. As a participant from North Wales said: ‘Coming here […] watching people growing, their journeys, is kind of, it's partly a reminder, partly reward, I get the reward feeling […] from assisting another person's journey in some […] small way’ [52, p. 7]. Persons in LTR described the importance of giving something back to society and to others; giving back was perceived as key to sustained recovery [44, 53]. Participants discussed how they welcomed the responsibility of being helpful through voluntary work [37].

## Discussion

**Figure 2:**
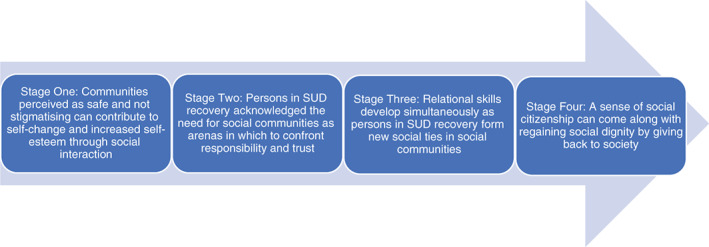
A model to guide research into social recovery from a first‐person perspective and how social communities support these processes. SUD, substance use disorder.

This is the first metasynthesis investigating the experiences of persons in SUD LTR in respect of connecting with social communities. Based on our findings, we propose a four‐stage model to guide the research on social recovery from a first‐person perspective and how social communities support this process (see Figure 2).

This metasynthesis underscores the need for a safe and non‐stigmatising environment. Previous research shows that the high degree of stigma and self‐stigma associated with having a SUD can be an obstacle to developing interpersonal relationships [16, 17, 21]. Arenas in which one can feel safe and non‐stigmatised seem to be a prerequisite for developing interpersonal relationships and connecting with social communities.

There are three types of social communities that were perceived as safe and non‐stigmatising: (i) communities such as NA; (ii) religious communities; and (iii) social communities with which the persons concerned have maintained contact since a time prior to the period of substance use. Access to these communities presupposes that a person connects with an NA group, that a person is religious and/or seeking spirituality, or that a person maintained relationships with family and friends while they used substances. However, while recovery research has emphasised connecting with communities through shared activities [13], there is still a need for further research into persons in LTR who are not connected with communities such as NA, AA or religious communities [15].

Moreover, the analysis showed that participants stopped using NA communities after a while as the participants no longer saw them as relevant. The literature does not provide insight into whether they then connected with other arenas.

The analysis shows that social communities are arenas in which to confront responsibility and trust. Findings show that persons in LTR must acknowledge the need to connect with social communities. This is in line with the relational theoretical perspective on recovery, which regards interpersonal relationships and social contexts as prerequisites for recovery [6]. The communities are important arenas for learning, the development of self‐confidence and self‐change. Our findings correspond with that of other research on this topic [1, 2, 3], underscoring the importance of having access to social communities. This review is in line with other syntheses that emphasise connectedness, building personal relationships, and a sense of community belonging as facilitators in the recovery process [4, 15]. At the same time, however, the cost of seeking out others in communities is felt to be high. Our findings show the early stage in the social recovery process to be characterised by a personal battle to overcome stigma, self‐stigma, and the fear of being vulnerable and to show others one's vulnerability. These are strains that seem to be understudied in the literature on LTR. LTR requires self‐acceptance and the overcoming of self‐stigma [38, 39]. When persons in recovery hesitate to seek out communities, this may be due to fear of the vulnerability itself [38, 55]. This finding is consistent with what one study has found as contributing to relapse [55]. Two of the main challenges that participants face is managing interpersonal relationships and building new supportive social communities [55]. The findings in our review show that acceptance of others leads to lessened discomfort and anxiety and a greater sense of trust in others [39, 48, 50]. The results of our review suggest that social interaction among persons in a social community causes a change in a person's experience of self‐acceptance and self‐worth.

This metasynthesis indicates that community is more than just an arena in which to be socialised into a role as a responsible citizen in the community. Indeed, it is also essential as an arena in which to regain one's social dignity, by way of voluntary work and giving back to society. Our findings support a review that argues that a sense of community belonging can be shaped by engaging in community participation [15]. Our review supports earlier research on overcoming stigma and self‐stigma by participating in activities that are experienced as meaningful [19]. Giving back is not limited to stage four of our model. The act of giving back made persons in recovery feel appreciated and valued. This was a rewarding feeling and the opposite of being stigmatised. Our results demonstrate that social communities can function as a springboard for getting involved in other groups and organisations in society. In this way giving back can help create a sense of citizenship. One may argue that social recovery is also a process through which persons in LTR experience the restoration of their social dignity.

### Strengths and limitations

The strength of this review is the international thematic convergence in the results section of the studies included. We did not set a time limit on the exclusion criteria, yet most of the articles included in this review have been published within the last 3 years. There are also limitations to our review that we would like to draw attention to. In this field of knowledge, much of the research has been concentrated on the NA/AA communities. What is known of the experience in other social communities is limited. Second, the fact that we found as many articles by searching manually as by way of a systematic search may indicate that our keywords were not sufficiently comprehensive. Third, there may be studies in languages other than English that would have provided information about affiliation with communities in addition to those that we found. Finally, another limitation is the inclusion of publications that have different operationalisations of the concept of LTR. Some of the studies use total abstinence, others use abstinence from the prime substance, and others use self‐reported recovery time. We have operationalised recovery from a SUD as abstinence from drugs for at least 12 months; however, a definition that focuses on the importance of ‘people's ability to lead full and contributing lives as active citizens’ addresses more of the complexity associated with LTR [22, p. 360]. There is ongoing conceptual discussion of what LTR is, and this is a conversation that needs to be continued.

### Clinical implications

Recovery within SUD is heavily influenced by the communities with which a person is involved. Our model can contribute to the conversation about how existing treatment and recovery support models can support persons within the social inclusion process. The pivotal role of the social community as identified in this review underscores the importance of recognising how difficult it is for persons in SUD recovery to connect with social communities. The results indicate the importance of investigating one's existing social network and facilitating and supporting persons in establishing new friends and connecting with safe and non‐stigmatised communities during LTR.

## Conflict of Interest

The authors have no conflicts of interest.
